# Rifaximin-Induced Rhabdomyolysis

**DOI:** 10.7759/cureus.104091

**Published:** 2026-02-22

**Authors:** Samuel Acheampong, Chidimma Udoyeh, Prince Darko, Eunice K Omeludike

**Affiliations:** 1 Internal Medicine, Piedmont Athens Regional Medical Centre, Athens, USA

**Keywords:** acute kidney injury, creatine kinase, rhabdomyolysis, rifaximin, statin use

## Abstract

Rifaximin is a non-absorbable oral antibiotic widely used for the management of traveler's diarrhea, hepatic encephalopathy, and irritable bowel syndrome diarrhea type. Although rifaximin is generally tolerated, rare cases of rhabdomyolysis have been reported, particularly in patients with cirrhosis with and without statin therapy. We reported a case of a 62-year-old male with a history of liver cirrhosis (ascites), hepatocellular carcinoma, and coronary artery disease (post coronary artery bypass grafting (CABG)) who presented with abdominal distension, generalized weakness, proximal muscle pain, and dark urine, with five years of atorvastatin medication without side effects. He was initiated on rifaximin (two weeks) for hepatic encephalopathy, along with lactulose, before experiencing proximal muscle pain and generalized weakness. This article will highlight our patient who developed rhabdomyolysis after initiating rifaximin with long-term use of statin, the trends of creatine kinase till resolution, and the treatment instituted.

## Introduction

Rhabdomyolysis is a condition characterized by the rapid breakdown of skeletal muscle tissue, releasing intracellular components, such as myoglobin, creatine kinase (CK), potassium, and phosphate into the bloodstream [1]. It can result from trauma, strenuous exercise, infections, alcohol, and medications - notably statins, which can cause drug-induced muscle damage. While rare, drug-drug interactions significantly increase the risk, particularly among individuals with metabolic myopathies and advanced liver disease [2]. Clinical signs mostly include muscle pain, muscle weakness, and dark-colored urine [3]. Rhabdomyolysis can be diagnosed in a patient with a probable history, significant elevation of CK, and acute kidney injury [4].

## Case presentation

Presentation and history

A 62-year-old male with a history of decompensated alcoholic liver cirrhosis with ascites, hepatocellular carcinoma, and coronary artery disease status, post triple vessel coronary artery bypass graft (CABG), presented with complaints of abdominal distention, as well as generalized weakness, muscle pain, and dark urine. Our patient developed generalized weakness and proximal muscle pain after rifaximin and lactulose therapy were initiated for hepatic encephalopathy. Two weeks prior to presentation, rifaximin 550 mg twice daily was initiated for hepatic encephalopathy in combination with lactulose. Shortly thereafter, the patient developed progressive muscle symptoms and was found to have markedly elevated CK levels consistent with rhabdomyolysis.

Physical examination

The patient was not in acute respiratory distress and was conscious and alert. He was hemodynamically stable, with blood pressure (BP) of 150/80 mmHg, pulse rate of 68 beats per minute, temperature of 36 °C, and respiratory rate of 16 cycles per minute (cardiopulmonary stable and abdominal distension with ascites). He had diffuse muscle tenderness, his motor strength was 5/5 in all extremities, and no asterixis was observed. His reflexes and tone were normal.

Laboratory results

His complete blood count revealed a white cell count of 5.0 × 10⁹/L, hemoglobin of 13.9 g/dL, and platelet count of 108 × 10⁹/L. Basic metabolic panel showed sodium of 137 mEq/L, potassium of 3.9 mEq/L, and creatinine of 1.33 mg/dL. Initial CK was 29,000 U/L, while anti-smooth muscle, anti-ribonucleoprotein (RNP), anti-Scl-70 immunoglobulin (IgG), anti-histone, anti-Jo-1 IgG, and anti-3-hydroxy-3-methylglutaryl-coenzyme A (HMG-CoA) reductase antibodies were all negative.

Hospital course, management, and outcome

The patient was admitted and managed for rhabdomyolysis and acute kidney injury.

Intervention: Statin was stopped on day one, and the patient was started on aggressive fluid at a rate of 300 mL/h, given significantly elevated CK for management. He also received intravenous Lasix to counterbalance volume overload.

**Figure 1:**
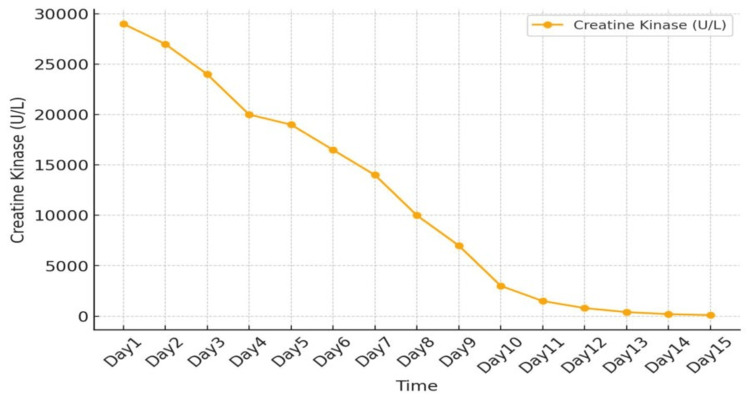
Trend of creatine kinase on days 1-15.

**Figure 2:**
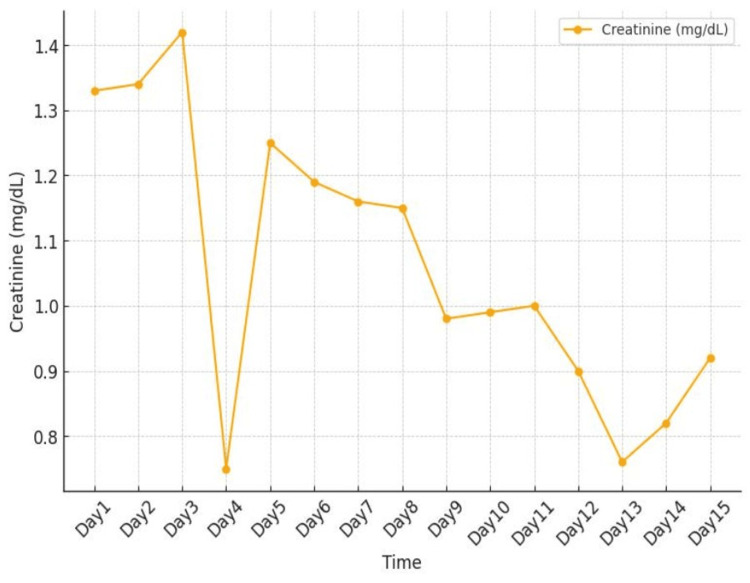
Trend of creatinine on days 1-15.

Rifaximin was discontinued, and the patient was managed with supportive care, leading to gradual normalization of CK levels and symptom resolution. Clinicians should maintain a high index of suspicion for rhabdomyolysis in this population and consider early recognition and monitoring when initiating rifaximin. Figure 1 presents the trend of CK on days 1-15. Figure 2 presents the trend of creatinine on days 1-15.

During day three of admission, the patient still had persistent muscle pain, and rifaximin was discontinued. The patient's symptoms have been ongoing for two weeks after starting rifaximin 550 mg twice daily. CK elevated significantly on the day of admission. CK levels gradually declined following discontinuation of rifaximin and normalized by hospital day 15.

## Discussion

Rhabdomyolysis and drug-induced muscle injury are well-established complications across many medications. Statins are one of the commonly implicated medications. Statins may cause muscle- and skeletal-related adverse events: myalgia, myositis, and rhabdomyolysis associated with CK elevation, about 10x above the normal limit, with or without acute kidney injury. Rhabdomyolysis induced by statins, while being uncommon, combining statin with other medications, which can lead to significant drug-drug interactions, thereby increasing the risk. The risk is noted to be increased with medications affecting cytochrome P-450, particularly the 3A4 isoenzyme [1,2].

The pathophysiology of rhabdomyolysis involves dysfunction of the myocyte Na-K ATPase channel and Ca-Na exchanger within the muscle. The initial insult leads to an elevation in intracellular calcium and water, resulting in cellular swelling and dysfunction [3]. The increased intracellular calcium levels trigger the activation of proteases and phospholipases. In addition to local inflammatory activities and increased production of free radicals, these mechanisms contribute to myocyte death and necrosis. This, in turn, releases myoglobin and creatine phosphokinase (CPK) into the circulation [3,4].

Rifaximin has been used for the treatment of travelers' diarrhea and irritable bowel syndrome [5]. While it is well-tolerated, there have been cases of rhabdomyolysis associated with its use, particularly in patients with cirrhosis, with or without concomitant statin use [6]. Although rifaximin is minimally absorbed in healthy individuals, patients with cirrhosis experience increased systemic exposure due to impaired hepatic metabolism and disrupted gut barrier function [7,8]. However, absorption is increased in individuals with severe cirrhosis, hepatic impairment that leads to systemic exposure to the drugs [9]. The rifampin component is implicated in the initiation of the mechanism of rhabdomyolysis induction, thereby adding to mitochondrial oxidative stress. This increase in oxidative stress increases the likelihood of rhabdomyolysis brought on by statins [10].

There are limited data on the incidence of rifaximin-induced rhabdomyolysis, but a review of the FDA MedWatch reporting system from 1977 to 2017 reported rhabdomyolysis as a side effect (health) of rifaximin in four patients. In a double-blind, phase 2, randomized controlled trial involving 44 patients, the safety of combining simvastatin and rifaximin in individuals with moderate-to-severe decompensated liver disease (Child-Pugh class B or C) was evaluated. Patients were allocated in a 1:1:1 ratio to receive either simvastatin 40 mg/day and rifaximin 1,200 mg/day, simvastatin 20 mg/day plus rifaximin 1,200 mg/day, or a placebo for both medications over a period of 12 weeks. Among the high-dose simvastatin group, comprising 16 patients, three (19%) developed rhabdomyolysis and liver injury (two Child-Pugh class C and one Child-Pugh class B) [11]. However, the safety profile of the lower simvastatin dose combined with rifaximin was comparable to that of the placebo. Most of the published literature concerning rifaximin-induced rhabdomyolysis involves concurrent use of statins. One case was documented in a patient with alcoholic liver cirrhosis and a Child-Pugh Score of class C. The patient in the case report developed rhabdomyolysis one month after initiating rifaximin 550 mg without concurrent statin use [11].

Even though our patient was on atorvastatin for an extended period, he never exhibited clinical or subclinical side effects associated with statins. We hypothesize that initiating rifaximin had a synergistic effect on a statin in this patient, subsequently leading to rhabdomyolysis following the start of rifaximin two weeks prior to admission.

## Conclusions

Rifaximin-associated rhabdomyolysis is a rare but potentially severe adverse effect. The risk increases in patients with advanced liver disease, especially when combined with statins. This case highlights an important association between rifaximin initiation and the development of rhabdomyolysis in a patient with advanced liver disease on long-term statin therapy, with clinical and biochemical improvement following drug discontinuation. In patients with cirrhosis presenting with unexplained myalgias, weakness, or acute kidney injury, clinicians should maintain a high index of suspicion for medication-induced rhabdomyolysis and systematically exclude alternative etiologies.
